# Complete genome sequences of two insect-specific flaviviruses

**DOI:** 10.1007/s00705-017-3552-5

**Published:** 2017-09-16

**Authors:** Jermilia Charles, Chandra S. Tangudu, Andrew E. Firth, Bradley J. Blitvich

**Affiliations:** 1 Department of Veterinary Microbiology and Preventive Medicine, College of Veterinary Medicine, Iowa State University, Ames, IA USA; 2 Department of Pathology, University of Cambridge, Cambridge, CB2 1QP UK

## Abstract

We determined the complete genomic sequences of two previously discovered insect-specific flaviviruses, Marisma mosquito virus (MMV) and Nanay virus (NANV), using a combination of high-throughput sequencing, reverse transcription-polymerase chain reaction, 5′ and 3′ rapid amplification of cDNA ends and Sanger sequencing. Complete polyprotein amino acid sequence alignments revealed that the closest known relatives of MMV and NANV are Donggang virus (89% identity, 95% similarity) and Nounané virus (53% identity, 70% similarity), respectively. Phylogenetic inference is in agreement with these findings. Potential programmed −1 ribosomal frameshifting sites were bioinformatically identified in the genomes of both viruses.

Viruses in the genus *Flavivirus* (family *Flaviviridae*) can be divided into three distinct groups based on their host ranges and mode of transmission [2]. Dual-host flaviviruses are transmitted horizontally between hematophagous arthropods (i.e. mosquitoes and ticks) and vertebrate hosts. Viruses in the other two groups possess vertebrate-specific or insect-specific host ranges. Insect-specific flaviviruses (ISFs) are further divided into classical ISFs (cISFs) and dual-host affiliated flaviviruses (dISFs). Viruses in the dISF group phylogenetically affiliate with dual-host flaviviruses despite their apparent insect-restricted phenotypes. Examples of dISFs include Marisma mosquito virus (MMV) and Nanay virus (NANV) in addition to Barkedji virus (BJV), Chaoyang virus (CHAOV), Donggang virus (DONV), Ilomantsi virus (ILOV), Lammi virus (LAMV), Nhumirim virus (NHUV) and Nounané virus (NOUV). MMV was originally isolated from *Ochlerotatus caspius* in Italy in 2001 to 2007 [17]. NANV was originally isolated from *Culex* (*Melanoconion*) *ocossa* in Peru in 2009 [6]. All these dISFs have had most, if not all, of their genomes fully sequenced aside from MMV and NANV for which partial envelope and/or NS5 gene sequence data are available. The objective of this study was to fully sequence the genomes of MMV and NANV.

MMV (isolate HU4528/07) and NANV (isolate PRD316/PER/09) were obtained from the World Reference Center for Emerging Viruses and Arboviruses at the University of Texas Medical Branch in Galveston, TX. MMV and NANV had been passaged four and at least five times, respectively in C6/36 (*Aedes albopictus*) cells prior to receipt and each virus underwent an additional passage in C6/36 cells at Iowa State University. Total RNA was extracted using Trizol Reagent (Invitrogen, Carlsbad, CA) and RNA was fragmented using RNase III and assessed for quality using an Agilent 2100 Bioanalyzer (Agilent, Santa Clara, CA). Libraries were constructed using the Ion Total RNA-Seq Kit v2 (ThermoFisher, Carlsbad, CA) and barcoded using the Ion Xpress™ RNA-Seq Barcode 1-16 Kit (ThermoFisher). Libraries were assessed for quality and analyzed at the Genomic Technologies Facility at Iowa State University using an Ion Proton Sequencer (ThermoFisher). All ion-Torrent reads were mapped to *Ae. albopictus and Ae. aegypti* genomes using Bowtie 2 [13]. Unmapped reads were analyzed using the sortMeRNA program [12] to remove rRNA-related reads. All remaining reads with Phred values ≥33 were subjected to *de novo* SPAdes (ver 3.5.0) assembly [1]. Contigs were mapped to a reference flavivirus genome using LASTZ [9]. Alignment files were manually verified on TABLET [16]. Reverse transcription-polymerase chain reaction and Sanger sequencing were used to close gaps and confirm the accuracy of assembled contigs. The 5′ and 3′ ends of each genome were identified using 5′ and 3′ rapid amplification of cDNA ends, respectively. Briefly, a DNA adaptor (5′-rApp/TGGAATTCTCGGGTGCCAAGGT/ddC-3′) was ligated to the viral genomic and anti-genomic RNAs using T4 RNA ligase (New England BioLabs, Ipswich, MA). Complementary cDNAs were created using SuperScript III (Invitrogen) and an adapter-specific primer. PCRs were performed using adapter- and gene-specific primers, and amplicons were subjected to Sanger sequencing.

The complete genome of MMV consists of 10,848 nt. (Genbank Accession No. MF139576) and contains a 5′ untranslated region (UTR) of 119 nt., a long open reading frame (ORF) of 10,353 nt., and a 3′ UTR of 376 nt (Figure 1a). The predicted amino acid sequence of the MMV polyprotein was aligned to other amino acid sequences in the Genbank database revealing that the closest known relatives of MMV are DONV (89% identity, 95% similarity) and ILOV (71% identity, 83% similarity). The complete genome of NANV is slightly smaller (10,804 nt; Genbank Accession No. MF139575) and contains a 5′ UTR of 106 nt., a long ORF of 10,299 nt., and a 3′ UTR of 399 nt (Figure 1b). Amino acid sequence alignments revealed that the closest known relatives of NANV are NOUV (53% identity, 70% similarity) and NHUV (52% identity, 69% similarity).

**Fig. 1:**
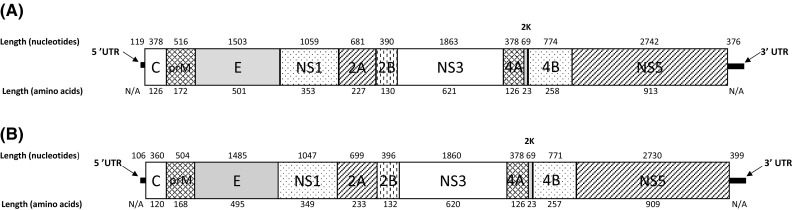
Schematic representation of each flavivirus genome and polyprotein. Genomic organization of (A) Marisma mosquito virus and (B) Nanay virus. Lengths of the 5’ and 3’ untranslated regions as well as the structural and nonstructural protein genes are drawn to scale

The phylogenetic placements of MMV and NANV, relative to other members of the *Flavivirus* genus, were assessed using the Bayesian Markov chain Monte Carlo based method implemented in MrBayes [15]. Complete polyprotein amino acid sequences were aligned using MUSCLE [5] and a phylogenetic tree was constructed using MrBayes (Figure 2). MMV is most closely related phylogenetically to DONV. Both viruses belong to a distinct clade that also includes CHAOV, LAMV and ILOV. NANV is most closely related phylogenetically to NOUV and these two viruses belong to a distinct clade that also includes BJV and NHUV.

**Fig. 2:**
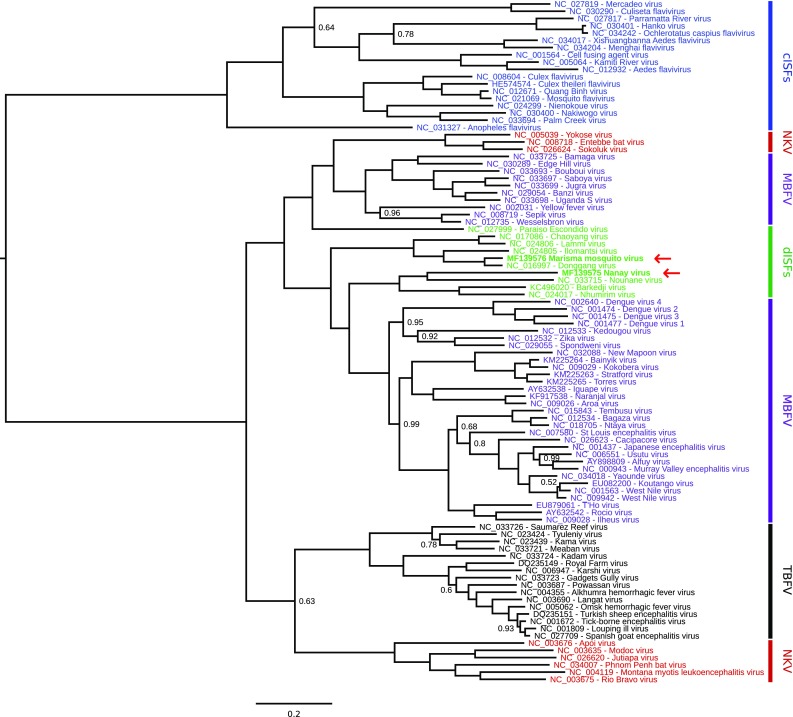
Phylogenetic tree for genus *Flavivirus*. Complete polyprotein amino acid sequences were aligned using MUSCLE [5]. Regions of ambiguous alignment were excised using Gblocks [3] with default parameters, after which 1604 amino acid positions were retained. A maximum likelihood phylogenetic tree was estimated using the Bayesian Markov chain Monte Carlo method implemented in MrBayes version 3.2.3 [15] sampling across the default set of fixed amino acid rate matrices, with one million generations, discarding the first 25% as burn-in. The figure was produced using FigTree (http://tree.bio.ed.ac.uk/software/figtree/). The tree is midpoint-rooted, and nodes are labelled with posterior probability values where different from 1.00. Species names are color-coded as follows: cISFs—blue; dISFs—green; no known vector (NKV) flaviviruses—red; mosquito/vertebrate flaviviruses—purple; tick/vertebrate flaviviruses—black

The sequences of MMV and NANV were inspected for potential −1 ribosomal frameshifting (−1 PRF) motifs because several groups of flaviviruses, including dISFs, appear to utilize −1 PRF during translation of their genomic RNA [2, 7, 10, 14]. Such frameshifting occurs at specific sites which normally comprise a slippery heptanucleotide sequence and a 3′-adjacent RNA structure. In eukaryotes, the consensus motif for the slippery heptanucleotide is X_XXY_YYZ, where XXX represents any three identical nucleotides although a number of exceptions occur (such as GGA), YYY represents AAA or UUU, Z is A, C or U, and underscores represent codons in the original reading frame. The 3′ RNA structure is normally a stem-loop or pseudoknot and is separated from the shift site by a spacer region of 5–9 nt. PRF has been predicted to occur in the NS2B region of CHAOV, DONV, LAMV, and ILOV [2, 10] and the ability of the identified motifs to stimulate −1 PRF has been verified in reporter constructs [8]. MMV contains a conserved G_GAU_UUU shift site sequence followed by a predicted RNA stem-loop structure in the NS2B region, as previously described for CHAOV, DONV and LAMV (Figure 3a). As noted previously, the −1 frame ORF varies considerably in length from six codons in DONV to 107 codons in CHAOV and LAMV. Similarly to DONV, MMV has a six-codon ORF.

**Fig. 3:**
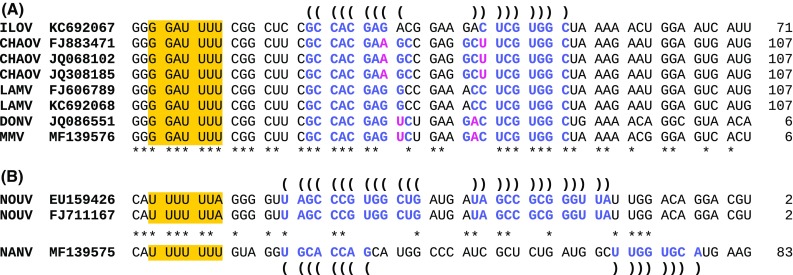
Predicted sites of ribosomal frameshifting. (a) Marisma mosquito virus and other members of the Chaoyang/Lammi/Donggang/Ilomantsi clade have a conserved G_GAU_UUU slippery heptanucleotide (orange highlight) followed by a predicted RNA stem-loop structure (blue letters) in the NS2B region. Compensatory substitutions (i.e. paired substitutions that preserve the predicted base-pairings) are indicated in pink. Parentheses indicate the predicted base-pairings. Numbers in the last column show the length of the −1 frame ORF. (b) A potential shift site (orange highlight) and adjacent RNA stem-loop structure (blue letters), conserved in location but not sequence, in the NS2A region of Nanay virus and Nounane virus

The genome of NANV harbors a U_UUU_UUU potential shift site that aligns with the U_UUU_UUA shift site previously proposed in NOUV (Figure 3b), and tested in reporter constructs [8]. While the NOUV shift site is followed by a compact 13-bp stem-loop, the potential shift site in NANV is followed by a more extended potential stem-loop. Nonetheless, the stem-loop has a stable base (seven consecutive Watson-Crick pairs, four being G:Cs) and is within the critical 5–9 nt. separation from the shift site. While the simple presence of a shift site and potential RNA structure should be viewed with caution (as they may occur spuriously, and not all RNA structures have the correct geometry to efficiently stimulate frameshifting [4]), the conservation of such features at a similar genomic location between related species lends weight to −1 PRF predictions. Thus, it seems plausible that NOUV and NANV may represent another group of flaviviruses that utilize −1 PRF.

In summary, we report the complete genome sequences of two previously discovered ISFs, MMV and NANV, and provide bioinformatic evidence that both viruses utilize –1 PRF. It remains to be proven whether dISFs evolved from dual-host flaviviruses or are themselves the precursors but the former theory has been favored [11]. A rapidly growing number of ISFs have been discovered in recent years, and the availability of complete genome sequence data will allow for more robust comparative genomic studies between dual- and single-host flaviviruses and could, ultimately, provide novel insight into the evolutionary mechanisms that condition their differential host ranges and transmissibilities.
